# Precocious deposition of perineuronal nets on Parvalbumin inhibitory neurons transplanted into adult visual cortex

**DOI:** 10.1038/s41598-018-25735-8

**Published:** 2018-05-10

**Authors:** Karen P. Bradshaw, Dario X. Figueroa Velez, Mariyam Habeeb, Sunil P. Gandhi

**Affiliations:** 1 Department of Neurobiology and Behavior, University of California, Irvine, CA 92697-4550 USA; 2 Center for the Neurobiology of Learning and Memory, University of California, Irvine, CA 92697-4550 USA

## Abstract

The end of the critical period for primary visual cortex (V1) coincides with the deposition of perineuronal nets (PNN) onto Parvalbumin (PV) inhibitory neurons. Recently, we found that transplantation of embryonic inhibitory neurons into adult V1 reinstates a new critical period. Here we used Wisteria Floribunda Agglutinin (WFA) staining to compare the deposition of PNNs onto neurons during normal development and following transplantation at equivalent cell ages. In accord with previous findings, PV and PNN expression increases from negligible levels at postnatal day 14 (P14) to mature levels by P70. In contrast to P14, PNNs are found on transplanted PV neurons by 21 days after transplantation and persist to 105 days after transplantation. This precocious deposition was specific to PV neurons and excluded transplanted neurons expressing Somatostatin. Notably, the onset of PV expression in transplanted inhibitory neurons follows the timing of PV expression in juvenile V1. Moreover, transplantation has no discernible effect on host PNNs. The precocious deposition of PNNs onto transplanted PV neurons suggests that PNN expression identified by WFA does not reflect neuronal maturity and may be an inaccurate marker for transplant-induced plasticity of cortical circuits.

## Introduction

The critical period for binocular vision is a time of heightened experience-dependent plasticity in primary visual cortex^1,2^. The maturation of GABAergic Parvalbumin-expressing (PV) inhibitory neurons in primary visual cortex has been linked to the onset of the critical period^3–7^. During postnatal development, perineuronal nets (PNNs) appear on PV inhibitory neurons and reach mature levels by the end of the critical period^8–13^. The disruption of PNNs and associated signaling in adult animals has been shown to restore visual cortical plasticity^9,10,14–18^. These findings suggest that the deposition of PNNs onto PV inhibitory neurons applies the brakes to critical period plasticity.

Recently, we and others have shown that the transplantation of embryonic inhibitory neurons from the medial ganglionic eminence opens a new critical period plasticity in both juvenile mice^19,20^ as well as adult recipients^21,22^. Transplanted inhibitory neurons from the medial ganglionic eminence reactivate critical period plasticity whereas inhibitory cells from the caudal ganglionic eminence do not^21–23^. In addition to reactivating ocular dominance plasticity, inhibitory neuron transplantation reverses visual acuity deficits brought on by early life visual deprivation^21^.

Transplanted inhibitory neurons recapitulate several developmental programs tied to cellular age. Cell death in transplanted inhibitory neurons follows a chronological developmental program^24^. Similarly, orientation selectivity in transplanted PV inhibitory neurons matures at the same cell age as for normally developing inhibitory neurons^25^. Moreover, transplantation creates a new critical period that occurs at a time when the donor animal would have had its critical period^21^. However, some developmental programs such as the maturation of membrane excitability have been found to mature more rapidly in transplanted cells than their host counterparts^26^.

Inhibitory neuron transplantation gives us a unique opportunity to assess the role of PNN deposition in closing the critical period for visual cortex. In this study, we examined the deposition of PNNs onto transplanted inhibitory neurons at three intervals relative to the transplant-induced critical period: before (21 days after transplantation), during (35 days after transplantation), and after (105 days after transplantation). We were guided by the hypothesis that a cell intrinsic developmental program governs the reactivation of cortical plasticity by transplanted inhibitory neurons. We found that the onset of PV expression in the transplanted cell population mirrors the normal timing found in developing cortex. Surprisingly, however, transplanted PV inhibitory neurons precociously acquire PNNs that remain stable over the course of the induced critical period. We also found that PNNs located on host inhibitory neurons are not perturbed by transplantation. Together, our results indicate that the timing of PNN deposition occurs more rapidly onto transplanted inhibitory neurons than anticipated. These findings challenge the hypothesis that PNN deposition alone is responsible for regulating the critical period.

## Results

### PNN deposition during postnatal development

**Figure 1:**
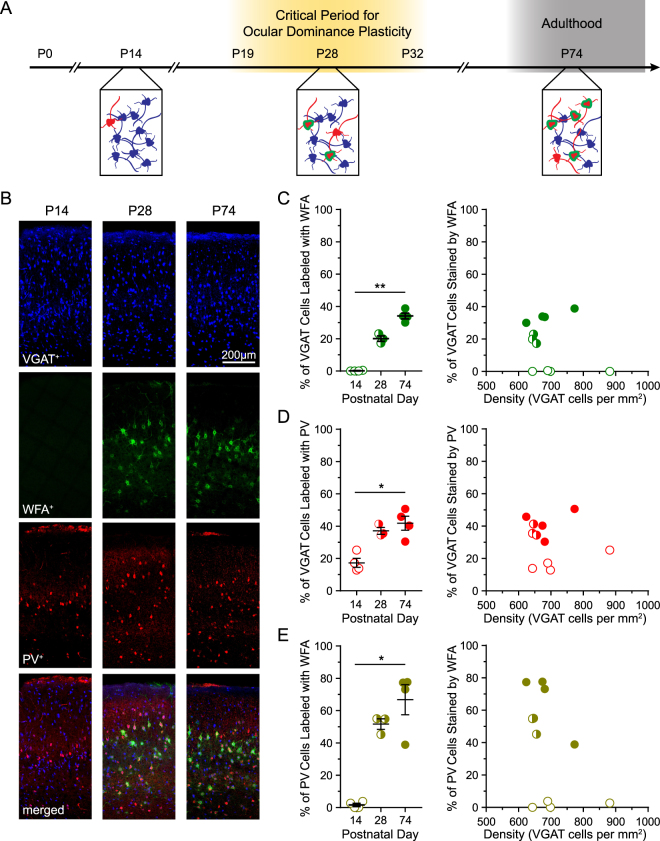
Deposition of PNNs onto PV inhibitory neurons in developing visual cortex. (**A**) Schematic timeline of PNN (green) development onto developing PV inhibitory neurons (red). At postnatal day 14 (P14), PNNs are absent and few inhibitory neurons (blue) express PV. By P28, at the height of the critical period, PNNs appear on some PV neurons. By P74, in adulthood, the density of PNNs has reached a mature level and most inhibitory PV cells carry PNNs. (**B)** Example sections from P14, P28, and P74 mice expressing a fluorescent protein in all VGAT neurons (blue). Wisteria Floribunda Agglutinin (green) lectin staining reveals the presence of PNNs. Nearly all PV cells (red) are co-localized with VGAT. (**C**) The percentage (left) of VGAT neurons co-labeled with WFA increases across postnatal age (P14 = 0.14 ± 0.12 (open), P28 = 20.11 ± 1.66 (half filled), P74 = 34.1 ± 1.82 (filled)) but is not correlated with VGAT cell density (right). (**D**) The percentage (left) of VGAT neurons expressing PV increases during postnatal development (P14 = 17.28 ± 2.79 (open), P28 = 37.09 ± 2.14 (half-filled), P74 = 41.79 ± 4.34 (filled)) but is not correlated with VGAT cell density (right). (**E**) percentage (left) of PV neurons stained with WFA increases across postnatal development (P14 = 1.63 ± 0.96 (open), P28 = 51.69 ± 3.24 (half-filled), P74 = 66.75 ± 9.34 (filled)). n_P14_ = 6,391 VGAT cells, 10 sections, 4 mice; n_P28_ = 7,159 cells, 11 sections, 3 mice; n_P63_ = 6,064 cells, 9 sections, 4 mice. Error bars represent s.e.m.

Previous studies have shown that GABAergic inhibitory neurons begin to acquire PNNs between P14 and P28 (Fig. 1A)^8,11,12^. To identify inhibitory neurons, we crossed mice harboring a Cre-dependent fluorescent protein reporter (tdTomato) with mice expressing Cre-recombinase under the control of the vesicular GABA transporter (VGAT) promoter. We first quantified the expression of PNNs on inhibitory neurons before (P14), during (P28), and after (P74) the juvenile critical period for ocular dominance plasticity (P19-P32)^1^ using a Wisteria Floribunda Agglutinin (WFA) stain (Fig. 1B).

As expected, we found a negligible (0.14%) percentage of inhibitory neurons carry perineuronal nets (PNNs) at P14 (Fig. 1C). By P28, the percentage of inhibitory neurons carrying nets increases to 20.11%, approximately half of that observed in P74 adults (34.1%, Fig. 1C). Importantly, the appearance of PNNs does not correlate with the density of inhibitory neurons as reflected by the density of VGAT containing cells (Fig. 1C). The developmental timeline of PNN expression in primary visual cortex we found is comparable to previous studies^11,27^.

Parvalbumin (PV) expression in primary visual cortex increases during the juvenile critical period^5,27,28^. We confirmed the developmental expression of PV by VGAT neurons (Fig. 1D). We found the percentage of VGAT neurons expressing PV significantly increases from 17.28% at P14 to 37.09% at P28 to 41.79% at P74 (Fig. 1D). Like PNN deposition, this increase in PV expression is not correlated with the density of VGAT cells (Fig. 1D). These analyses provide a timeline for PV expression in mouse visual cortex that closely aligns with previous studies^11,28–30^.

PNNs preferentially surround PV inhibitory neurons^5,8,11,31,32^. PNN deposition on PV neurons grows from virtually nothing at P14 (1.63%) to 51.69% at P21 and to 66.75% in adults (Fig. 1E). The presence of PNNs surrounding PV cells is not correlated to the density of VGAT cells (Fig. 1E). Our results confirm that PNN deposition on PV inhibitory neurons increases over the course of postnatal development^11,27^.

### Transplanted MGE cells disperse in adult visual cortex

**Figure 2:**
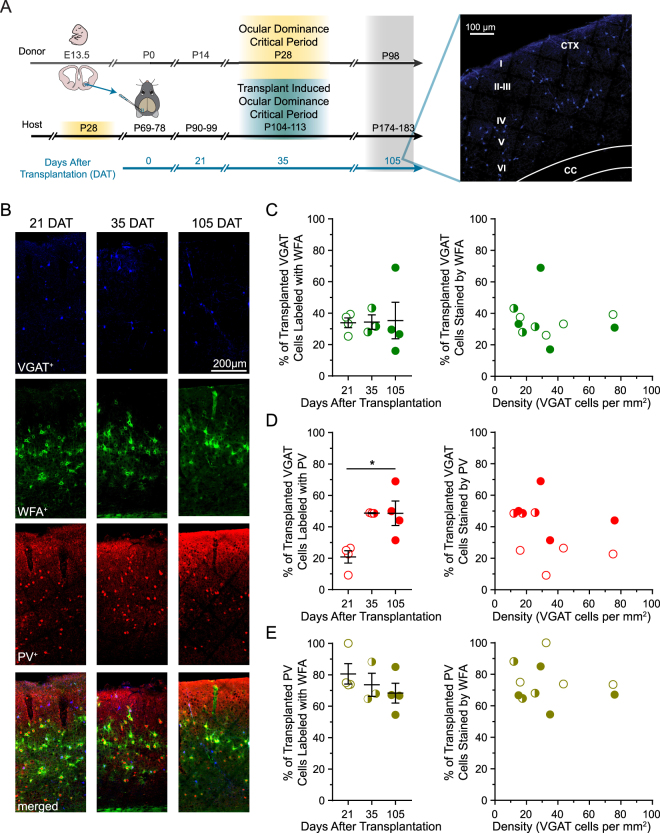
Precocious deposition of PNNs on transplanted PV inhibitory neurons. (**A**) Timeline of transplantation procedures (left) and coronal section 21 days after transplantation (right). Fluorescently-labeled cells from the medial ganglionic eminence of donors were dissected at embryonic day 13.5 and transplanted into adult visual cortex. Transplant recipients were perfused and analyzed 21, 35, and 105 days after transplantation (DAT) when the donor would have been P14, P28, and P98, respectively. Transplanted inhibitory neurons disperse across cortical layers I-VI (right). (**B**) Example sections of primary binocular visual cortex from transplant recipients at 21, 35, and 105 DAT. Transplanted VGAT neurons (blue) are stained with WFA (green) and PV (red). By 21 DAT, most transplanted inhibitory neurons that express PV are co-labeled with WFA. (**C**) Percentage of transplanted VGAT neurons stained by WFA (left) is similar among the three timepoints assessed (left: 21 DAT = 33.87 ± 3.12 (open), 35 DAT = 34.27 ± 4.59 (half-filled), 105 DAT = 35.27 ± 11.62 (filled)) and is not correlated with the density of transplanted cells (right). (**D**) In contrast, the percentage of transplanted VGAT neurons expressing PV (left) increases after 21 DAT (21 DAT = 20.84 ± 3.95 (open), 35 DAT = 48.71 ± 0.15 (half-filled), 105 DAT = 48.62 ± 7.81 (filled)). This increase in PV expression is not correlated with the density of transplanted cells (right). (**E**) Nonetheless, the percentage of transplanted PV neurons co-labeled with WFA (left) is stable across all timepoints assessed (21 DAT = 80.61 ± 6.47 (closed), 35 DAT = 73.65 ± 7.34 (half-filled), 105 DAT = 68.34 ± 6.27 (filled)). The density of transplanted neurons is not correlated with the percent of transplanted PV cells co-labeled with WFA (right). n_21DAT_ = 1,010 transplanted VGAT cells, 7 sections, 4 mice; n_35DAT_ = 343 transplanted VGAT cells, 7 sections, 3 mice; n_105DAT_ = 847 transplanted VGAT cells, 6 sections, 4 mice. Error bars represent s.e.m.

Transplantation of inhibitory neurons into adult visual cortex from the medial ganglionic eminence (MGE) introduces a new window of critical period plasticity^19–22^. If the deposition of PNNs terminate critical period plasticity, then more transplanted inhibitory neurons should carry perineuronal nets after the transplant-induced critical period^21^ has subsided, between 35 and 105 days after transplantation (DAT; Fig. 2A). To test this prediction, we harvested embryonic day 13.5 (E13.5) tissue from the MGE, a major source of cortical inhibitory neurons^33,34^. We then transplanted MGE precursors into sites bordering the binocular primary visual cortex using a physiological mapping procedure^21^. Transplanted inhibitory neurons were identified by fluorescence and appear to disperse throughout all layers of the adult primary visual cortex as previously reported^21^.

### Precocious deposition of PNNs on transplanted inhibitory neurons

We quantified the percentage of transplanted inhibitory neurons surrounded by PNNs before (21 DAT), during (35 DAT), and after the transplant-induced critical period (105 DAT). The youngest host age during which transplantation co-labeling was assessed is P90, well after the normal critical period (Fig. 2A). Surprisingly, by 21 DAT we found that deposition of PNNs on transplanted neurons is already 33.87% (Fig. 2C), comparable to those observed in non-transplanted adults (34.1%; Fig. 1C). More importantly, the percent of transplanted inhibitory neurons with PNNs is similar before (33.87%), during (34.27%), and after (35.27%) the transplant induced critical period (Fig. 2C). Moreover, the density of transplanted inhibitory neurons could not explain the percentage of transplanted cells carrying PNNs. These results reveal the precocious development of PNNs on transplanted inhibitory neurons.

### PV expression in transplanted cells is largely independent of host cell age

**Figure 3:**
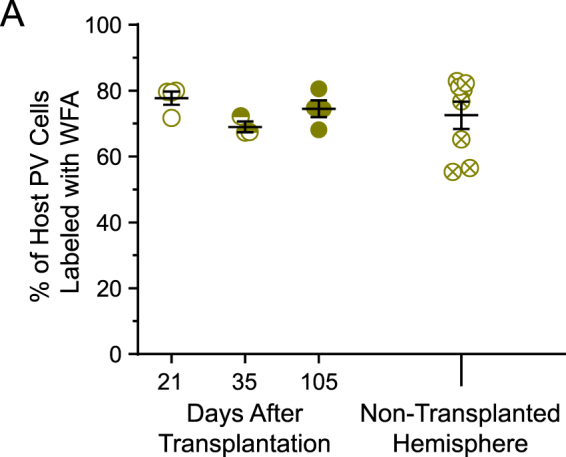
Transplantation does not disturb PNNs on host PV inhibitory neurons. (**A**) Host PV cells co-labeled with WFA was unaffected by transplantation and similar to the non-transplanted hemisphere (N-TH) (21 DAT = 77.7 ± 2.00 (open), 35 DAT = 68.97 ± 1.64 (half-filled), 105 DAT = 74.5 ± 2.54 (filled), N-TH = 72.54 ± 4.14 (crossed)). n_21DAT_ = 1,203 host PV cells, 7 sections, 4 mice; n_35DAT_ = 845 host PV cells, 7 sections, 3 mice; n_105DAT_ = 817 host PV cells, 6 sections, 4 mice; n_N-TH_ = 1,057 host PV cells, 10 sections, 8 mice. Error bars represent s.e.m.

Over the course of the normal critical period, we observed that PV expression more than doubles (Fig. 1D). To determine how transplanted inhibitory neurons develop PV expression we examined PV and PNN co-expression 21, 35, and 105 days after transplantation (Fig. 3). Like in normal development, PV expression is significantly lower in the transplanted inhibitory (VGAT) cell population before the induced critical period at 21 DAT (20.84%, Fig. 3D). Contrary to normal development, the number of transplanted cells expressing PV reaches adult levels by 35 DAT (48.71%) and remains stable through 105 DAT (48.62%). Nonetheless, the percentage of transplanted PV neurons at 21 DAT and 35 DAT are comparable with values observed at P14 and P28, respectively^11,28^. We did not find this increase in PV expression to be correlated to the density of transplanted inhibitory (VGAT) cells. Together, our findings suggest that PV maturation is largely cell-intrinsic and independent of host age.

### Transplanted cells acquiring PNNs are fated to express PV

In our developmental study, we observed a strong specificity of PNNs for PV inhibitory neurons (Fig. 1). To assess whether this specificity was present in transplanted neurons, we quantified the percent of transplanted PV neurons that carried PNNs at 21, 35, and 105 DAT (Fig. 3E). Surprisingly, the percentage of transplanted PV neurons bound to PNNs had already reached adult levels by 21 DAT (80.61%; Fig. 3E). This was in remarkable contrast to the almost non-existent percentage of PV inhibitory neurons bound to PNNs at P14 observed by our group (1.63%) and others^9,11^. The percentage of transplanted PV neurons with PNNs remained stable through 35 (73.65%) and 105 DAT (68.34%; Fig. 3E). Our findings suggest that PNNs precociously bind to transplanted PV neurons and remain on them throughout the course of the transplant-induced critical period.

### Transplantation does not degrade PNNs in host PV neurons

Recently, it was shown that MGE neurons transplanted in the adult amygdala degrade the PNNs on host inhibitory neurons during a period of transplant-induced plasticity^35^. Although the presence of PNNs on transplanted inhibitory neurons is similar from 21 and 105 DAT, transplantation into adult visual cortex may considerably alter host PNNs. To evaluate the possible degradation of host PNN in adult visual cortex, we quantified the percentage of host PV cells with PNNs in the transplanted hemisphere and compared those values to the non-transplanted hemisphere (Fig. 3). We found that the fraction host PV cells bound to PNNs in the transplanted hemisphere remains at adult levels (21 DAT = 77.7%, 35 DAT = 68.97%, 105 DAT = 74.5%) and is similar to the non-transplanted hemisphere (72.54%) (Fig. 3). These results suggest MGE transplantation does not degrade host PNNs.

**Figure 4:**
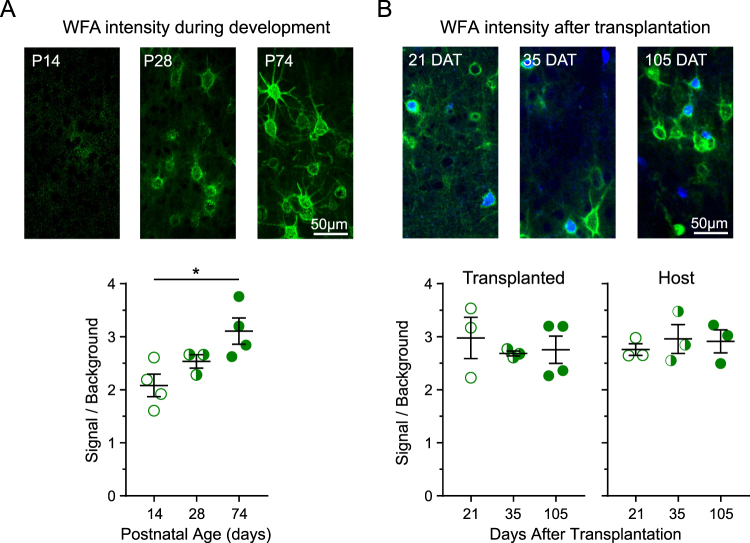
WFA staining intensity of PNNs surrounding host and transplanted cells does not depend on transplanted cell age. Quantification of WFA staining intensity of PNNs around PV cell bodies. The intensity of WFA fluorescence around the PV cell body (5 pixel thick region of interest) was normalized to the background by dividing its value by the staining intensity of an adjacent, unstained region. (**A**) Example sections stained with WFA at three ages spanning development (top). The signal to background ratio (bottom) increases during postnatal development (P14 = 2.08 ± 0.21 (open), P28 = 2.54 ± 0.13 (half-filled), P74 = 3.11 ± 0.25 (filled)). (**B**) Example sections of transplanted and host cells surrounded co-labeled with WFA 21, 35, and 105 DAT (top). The WFA signal to background ratio for transplanted cells (bottom left) was similar at the three timepoints assessed (21 DAT = 2.98 ± 0.39 (open), 35 DAT = 2.69 ± 0.05 (half-filled), 105 DAT = 2.76 ± 0.26 (filled)). Transplantation did not alter host WFA signal to background ratio (bottom right; 21 DAT = 2.76 ± 0.11 (open), 35 DAT = 2.96 ± 0.27 (half-filled), 105 DAT = 2.91 ± 0.22 (filled)). Normal cell development: n_P14_ = 40 cells, 8 sections, n = 4 mice; n_P28_ = 165 cells, 11 sections, 3 mice; n_P74_ = 13 cells, 4 mice. Transplanted neurons: n_21DAT_ = 48 cells, 6 sections, n = 4 mice; n_35DAT_ = 43 cells, 7 sections, 3 mice; n_105DAT_ = 78 cells, 6 sections, 4 mice. Host neurons: n_21DAT_ = 47 cells, 6 sections, n = 3 mice; n_35DAT_ = 54 cells, 7 sections, 3 mice; n_105DAT_ = 81 cells, 6 sections, 3 mice. Error bars represent s.e.m.

It was possible that the precocious PNNs we observed were ill-formed and immature. We investigated this possibility by first examining the intensity of WFA for PNNs in normal development (Fig. 4A). It has been observed that the intensity of WFA staining increases as PNNs mature^36^. We quantified the WFA fluorescence intensity of the PNNs surrounding the cell body of PV inhibitory neurons and normalized it to the average intensity value of an adjacent unstained area. As expected, we found that WFA intensity around PV neurons significantly increases across postnatal age from P14 (2.08) to P28 (2.54) and to P74 (3.11), increasing by more than 50% over the course of the juvenile critical period.

**Figure 5:**
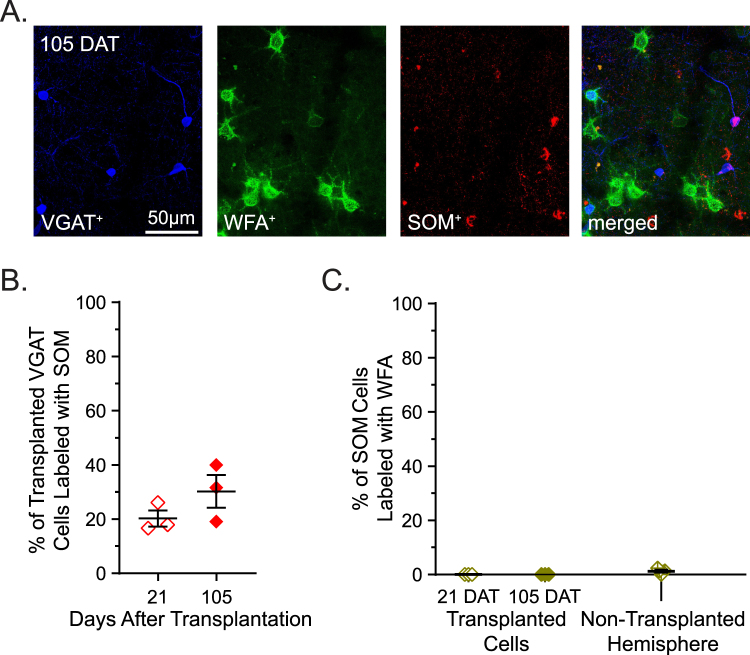
PNNs are not associated with transplanted SOM inhibitory neurons. (**A**) Example sections of primary visual cortex from transplant recipients at 21, 35, and 105 DAT. Transplanted VGAT neurons (blue) are stained with WFA (green) and SOM (red). (**B**) The percentage of transplanted SOM cells 21 and 105 DAT (21 DAT = 30.23 ± 6.07 (open), 105 DAT = 20.23 ± 2.95 (filled)). (**C**) Transplanted and host SOM cells are not co-labeled with WFA (21 DAT = 0 ± 0 (open), 105 DAT = 0 ± 0 (filled), N-TH = 1.22 ± 0.38 (crossed)) n_21DAT_ = 105 VGAT cells, 6 sections, n = 3 mice; n_105DAT_ = 116 VGAT cells, 3 sections, 3 mice; n_N-TH_ = 421 SOM cells, 5 slices, 5 mice. Error bars represent s.e.m.

Next, we tested whether the PNNs observed on transplanted PV inhibitory neurons were mature (Fig. 4B). We found that WFA staining intensity surrounding transplanted PV neurons remains consistent across the cell ages tested (21 DAT = 2.98, 35 DAT = 2.69, 105 DAT = 2.76) (Fig. 5B). We observed comparable levels of WFA staining in host PV neurons in the transplanted hemisphere regardless of the time after transplantation (21 DAT = 2.76, 35 DAT = 2.96, 105 DAT = 2.91) (Fig. 4B). The consistency of WFA staining intensity across groups further supports our observation that PNN deposition does not depend upon the age of the transplanted inhibitory cells.

### Transplanted Somatostatin cells do not acquire PNNs

It has been shown that transplanted Somatostatin (SOM) cells alone reactivate plasticity in visual cortex^20^. Therefore, it may be that PNNs are deposited onto transplanted SOM cells and terminate the transplant-induced period of cortical plasticity. To test this hypothesis, we stained transplanted tissue 21 and 105 DAT for SOM and PNNs (Fig. 5A). During normal development, the number of cells expressing SOM reaches adult levels by P14^28^. Similarly, we found that transplanted cells labeled with SOM 21 DAT (30.23%) is not different from 105 DAT (20.23%) (Fig. 5B). More importantly, we did not find SOM cells surrounded by PNNs in either transplanted cells (21 DAT = 0%, 10 DAT = 0%) or in host cells of the non-transplanted hemisphere (1.22%) (Fig. 5C and Table 1).

**Table 1:** Statistical Analysis.

	Group	Kruskal-Wallis		Mann-Whitney			Spearman	
		H	p	Subgroup test	U	p	r	p
Figure 1	VGAT cells co-labeled with WFA	8.95	0.0005	P14 v P28	0	0.0571	−0.04338	0.8986
				P14 v P74	0	0.0286		
				P28 v P74	0	0.0571		
	VGAT cells co-labeled with PV	7.212	0.0090	P14 v P28	0	0.0571	−0.2506	0.4548
				P14 v P74	0	0.0286		
				P28 v P74	4	0.6286		
	PV cells co-labeled with WFA	7.511	0.0059	P14 v P28	0	0.0571	−0.4132	0.2041
				P14 v P74	0	0.0286		
				P28 v P74	3	0.4000		
Figure 2	Transplanted VGAT cells co-labeled with WFA	0.7993	0.5758	21 DAT vs 35 DAT	6	>0.9999	−0.287	0.3897
				21 DAT vs 105 DAT	6	0.6857		
				35 DAT vs 105 DAT	4	0.6286		
	Transplanted VGAT cells co-labeled with PV	7	0.0159	21 DAT vs 35 DAT	0	0.0571	−0.4364	0.1826
				21 DAT vs 105 DAT	0	0.0286		
				35 DAT vs 105 DAT	6	>0.9999		
	Transplanted PV cells co-labeled with WFA	2.598	0.3061	21 DAT vs 35 DAT	3	0.4000	−0.1545	0.6538
				21 DAT vs 105 DAT	3	0.2000		
				35 DAT vs 105 DAT	4	0.6286		
Figure 3	Host WFA cells co-labeled with PV Normal Host Development	2.213	0.5609	N-TH vs 21 DAT	15	0.9333	—	—
				N-TH vs 35 DAT	9	0.6303		
				N-TH vs 105 DAT	14	0.8081		
Figure 4	WFA intensity during development	7.144	0.0097	P14 v P28	1	0.1143	—	—
				P14 v P74	0	0.0286		
				P28 v P74	2	0.2286		
	WFA intensity after transplantation (Transplanted)	0.1636	0.9410	21 DAT vs 35 DAT	3	0.7000	—	—
				21 DAT vs 105 DAT	6	>0.9999		
				35 DAT vs 105 DAT	6	>0.9999		
	WFA intensity after transplantation (Host)	0.2667	0.9286	21 DAT vs 35 DAT	4	>0.9999	—	—
				21 DAT vs 105 DAT	3	0.7000		
				35 DAT vs 105 DAT	4	>0.9999		
Figure 5	Transplanted VGAT cells co-labeled with SOM	—	—	21 DAT vs 105 DAT	1	0.2000	—	—
	Transplanted SOM cells	6.479	0.0515	N-TH vs 21 DAT	1.5	0.1250	—	—
	co-labeled with WFA			N-TH vs 105 DAT	1.5	0.1250	—	—

## Discussion

Recently we found that the transplantation of embryonic inhibitory neurons creates a new period of heightened plasticity in adult visual cortex^21^. The end of critical period plasticity has been linked to the deposition of perineuronal nets (PNNs) onto Parvalbumin-expressing (PV) neurons^8–13,37^. Therefore, we might predict that PNNs are deposited onto transplanted PV inhibitory neurons when transplant-induced plasticity subsides, more than 35 days after transplantation (35 DAT)^19,21^. Alternatively, transplantation could reactivate plasticity by degrading mature PNNs on host PV inhibitory neurons^35^. In this study, we discovered that PV inhibitory neurons transplanted into adult visual cortex acquire PNNs by 21 DAT, much sooner than expected. The level of PNN expression that we found on transplanted inhibitory neurons is comparable to adult levels and remains constant up to 105 DAT.

We also found that transplantation did not disturb the presence of PNNs on host neurons at any of the timepoints studied. A recent study found that inhibitory neurons transplanted into the adult basolateral amygdala induces a new critical period for fear erasure^35^. In contrast to our study of transplantation into adult visual cortex, Yang *et al*. show that transplantation reduces PNNs surrounding host neurons in the amygdala. The apparent discrepancy suggests that the mechanisms of critical period reactivation may be specific to the host circuit into which these cells integrate.

The maturation of PV inhibitory neurons plays a key role in critical period plasticity^2,3^. It is well known that PV expression in the primary visual cortex of mice increases dramatically between P14 and P28^27–30^. In contrast to the precocious deposition of PNNs on transplanted inhibitory neurons, we find that PV expression in transplanted inhibitory neurons more than doubles between 21 and 35 DAT, suggesting that PV expression is determined by cell age. The developmental timing of PV expression in transplanted inhibitory neurons agrees with other findings of cell-intrinsic programs in these cells such as developmental apoptosis^24^, orientation selectivity^25^, and reactivation of cortical plasticity^21^.

Previous studies have shown that PNNs preferentially bind to PV-expressing inhibitory neurons^5,11,31,32^. In our study, we find that PNNs associate with transplanted PV inhibitory neurons and not SOM neurons, respecting this close association. Nonetheless, the deposition reaches adult levels much earlier than expected suggesting that PNN deposition is not a cell-intrinsic process.

PNNs surrounding PV neurons may gate critical period plasticity by capturing key signaling factors^15,38^. The transcription factor Otx2 has been identified to associate with PNNs and regulate critical period plasticity in a positive feedback loop^12,15,29,39^. Perhaps transplanted PV inhibitory neurons acquire PNNs more rapidly than expected because the adult levels of Otx2 present in the host visual cortex are sufficient to stimulate PNN deposition. The conditional genetic deletion of Otx2^40^ from host animal may provide an experimental strategy to test the role of this factor in PNN deposition on transplanted cells.

To visualize PNNs, we used Wisteria floribunda agglutinin (WFA) staining. The maturation of PNNs has been associated with the intensity of WFA staining^36^. We found that the intensity of WFA staining on PV neurons increased from P14 to P28. In contrast, the intensity of WFA staining on transplanted PV neurons reached adult levels by 21 DAT, suggesting the precocious maturation of PNNs on these cells. Furthermore, the intensity of WFA staining on host PV neurons in the transplanted and non-transplanted hemisphere suggests that transplantation did not affect the maturity of PNNs in the host circuitry.

It is possible that the maturational state of PNNs are not adequately captured in our study by WFA staining. PNNs are composed of a multitude of proteoglycans including aggrecan and tenascin-R^38,41,42^. Although aggrecan and tenascin-R staining have similar developmental profiles as WFA staining^11^, other PNN components may better reflect the maturity of PNNs. Future studies on specific changes to the configuration of proteoglycans in PNNs surrounding transplanted neurons may reconcile our findings with the normal development of PNNs.

In this study, we find that for transplant-induced plasticity, PNN expression is an inaccurate marker for the plasticity of cortical circuits. Similar to normal juvenile plasticity, transplantation-induced plasticity is limited to a brief critical period, primarily affects deprived-eye responses, and shapes the spatial acuity of cortical responses^19–22^. Nonetheless, the mechanisms regulating transplant-induced plasticity may be distinct from the juvenile critical period. Future studies on how transplantation alters the cortical circuit may reveal plasticity mechanisms independent of PNNs^43–46^.

## Methods

All experiments were approved by the Institutional Animal Care and Use Committee at the University of California, Irvine (2011–2994) and were conducted according to the NIH guide for the Care and Use of Animals.

### Animals

Embryonic donor tissue and mice used to characterize postnatal development were produced by crossing the Cre-dependent tdTomato reporter line (Ai14, JAX 007914) with mice that express Cre recombinase under the control of the Vesicular GABA Transporter (VGAT) promoter (VGAT-ires-Cre, JAX 028862). Wild-type C57BL/6J mice (JAX 000664) were used as recipients for transplantation.

### Intrinsic Signal Imaging

As described previously^21^, intrinsic signal optical imaging was used to find the binocular visual cortex in host P69–78 day-old mice. Briefly, anesthesia was induced with 2.5% isofluorane and the dose was reduced 0.8% for intrinsic signal mapping. Mice were given intraperitoneal chlorprothixene injections. Mice were presented with a spatiotemporal noise stimulus that swept from −18° to 36° visual field elevation with a periodicity of 0.1 Hz. Fourier analysis of the red light reflection from brain revealed a retinotopic map of binocular visual cortex.

### Tissue Dissection

Bilateral medial ganglionic eminences were dissected from embryonic day 13.5 (E13.5) VGAT-Ai14 embryos using previously described methods^21^. Medial ganglionic eminence tissue was chilled in Leibovitz’s L-15 medium and loaded into a glass micropipette (~75 µm tip diameter, Wiretrol 5 microliter, Drummond Scientific Company) using a custom-designed hydraulic injection tool.

### Retinotopic Map-Guided Cell Transplantation

The retinotopic map obtained using intrinsic signal imaging was used to guide skull incisions medial and lateral to the binocular visual cortex. Donor cells were injected at a 45° angle to the cortical surface and advanced approximately 700 µm into the cortex. Three 15–20 nL injections were made in each of the two slits. After transplantation, the scalp was sutured, anesthesia was terminated, and the animal was placed on a warm heating pad for recovery.

### Histological Preparation and Cell Counting

Animals were transcardially perfused (4% in 1 × PBS) either 21 days (n = 4), 35 days (n = 3), or 105 days after transplantation (n = 4). Brains were removed, post-fixed, and cryopreserved in 30% sucrose. A freezing sliding microtome was used to slice the brains into 50 μm thick coronal sections (Microm HM450). The free-floating slices of tissue were stained and blocked for one hour at room temperature with 0.5% Triton-X (Sigma T8787) and 10% BSA (Fisher BP1600-100) in 1X PBS. Slices were incubated overnight at 4 °C with the primary antibodies rabbit-anti-RFP (tdTomato 1:1000, Abcam ab62341) and mouse-anti-PV (1:1000, Sigma P3088). The sections were washed three times in 1X PBS for five minutes. They were then incubated for two hours in 594 goat-anti-rabbit IgG (1:1000, Invitrogen) for VGAT cells, 647 goat-anti-mouse IgG1 (1:1000, Invitrogen) for PV cells, and Fluorescein labeled Wisteria Floribunda Agglutinin (WFA), Vector Labs) for PNN^+^ cells. The stained tissue was mounted on glass slides with Fluoroshield with DAPI. The sections were imaged with a confocal microscope (Leica SP8, 63X objective, N.A 1.4).

ImageJ was used to count the cells expressing VGAT, PV, and WFA in brain sections. The investigator was blinded to groups for PNN identification and counting. A PNN was positively identified if it appeared to robustly surround at least three-fourths of a cell body.

ImageJ was also used to assess the intensity of PNN staining (Image J ROI manager, version 1.51j, NIH). 5-pixel thick regions of interest were drawn on a randomly selected subset of identified PNNs. PNN intensity was normalized by dividing the average intensity value of the net by the average intensity value of the background. Background regions of interest were defined using the PNN’s region of interest applied to the dimmest neighboring background.

### Statistical Analyses

Kruskal-Wallis ANOVA was used as an omnibus test for significant differences among groups. A Mann-Whitney test with Bonferroni correction was used for pairwise comparisons. Statistical tests were performed using Prism version 7.02 (Graphpad).

Materials, data, and associated protocols are available upon request from the corresponding author.
